# Editorial Comments on PTHrP‐Producing Renal Cell Carcinoma Presenting as Rapidly Progressive Cognitive Impairment: A Case Report

**DOI:** 10.1002/iju5.70169

**Published:** 2026-03-16

**Authors:** Kento Morozumi

**Affiliations:** ^1^ Department of Urology Tohoku Medical and Pharmaceutical University Sendai Miyagi Japan

Hypercalcemia of malignancy (HCM) is a clinically important paraneoplastic syndrome in urologic oncology, particularly in renal cell carcinoma (RCC) [1]. Although PTHrP‐mediated hypercalcemia is well recognized, presentation with rapidly progressive cognitive impairment is uncommon and may delay diagnosis. In this context, the case report by Yano et al. [2] highlights that metabolic derangements can be a primary cause of acute neuropsychiatric symptoms in RCC.

The authors describe a 68‐year‐old woman with rapidly progressive cognitive decline who was diagnosed with sarcomatoid RCC complicated by severe PTHrP‐mediated hypercalcemia. Prompt normalization of serum calcium and marked cognitive recovery after radical nephrectomy demonstrate the reversibility of neurological symptoms once the paraneoplastic source is removed. This clinical course underscores an important diagnostic message: unexplained and rapidly progressive cognitive impairment should prompt evaluation for metabolic abnormalities, including hypercalcemia.

Several aspects of this report deserve attention. First, the absence of distant metastases despite markedly elevated PTHrP levels is notable. While hypercalcemia is often associated with advanced disease, this case illustrates that aggressive tumor biology, such as sarcomatoid differentiation, may drive excessive humoral factor production even in clinically localized RCC. Sarcomatoid features correlate with aggressive behavior and poor prognosis, emphasizing the importance of tumor biology beyond disease stage alone. Second, the report reinforces the prognostic relevance of hypercalcemia itself, an established adverse factor in major prognostic models for metastatic RCC, including the MSKCC and IMDC criteria [3, 4]. Although immunohistochemical confirmation of PTHrP expression and serial postoperative PTHrP measurements were limited, the markedly elevated serum PTHrP with suppressed intact parathyroid hormone and its rapid normalization after nephrectomy provide sufficient indirect evidence to support the diagnosis.

From a therapeutic perspective, this case highlights the enduring role of surgical intervention in selected patients with paraneoplastic syndromes. While systemic therapies, including immune checkpoint inhibitors, have transformed the management of advanced RCC, definitive local control remains essential for resolving paraneoplastic metabolic complications. Supportive measures such as hydration and bisphosphonates may provide temporary relief, but tumor‐directed therapy is required for sustained recovery [5].

This case highlights atypical PTHrP‐producing RCC and underscores evaluation and timely surgery for reversible paraneoplastic cognitive dysfunction.

## Funding

The author has nothing to report.

## Conflicts of Interest

The author declares no conflicts of interest.

## Data Availability

The data that support the findings of this study are available from the corresponding author upon reasonable request.

## References

[1] T. A. Guise and J. J. Wysolmerski , “Cancer‐Associated Hypercalcemia,” New England Journal of Medicine 386, no. 15 (2022): 1443–1451. 35417639 10.1056/NEJMcp2113128

[2] F. Yano , N. Sawada , N. Tanaka , et al., “PTHrP‐Producing Renal Cell Carcinoma Presenting as Rapidly Progressive Cognitive Impairment: A Case Report,” IJU Case Reports 9, no. 1 (2026): e70142. 41567516 10.1002/iju5.70142 PMC12819007

[3] R. J. Motzer , N. M. Tannir , D. F. McDermott , et al., “Nivolumab Plus Ipilimumab Versus Sunitinib in Advanced Renal‐Cell Carcinoma,” New England Journal of Medicine 378, no. 14 (2018): 1277–1290. 29562145 10.1056/NEJMoa1712126 PMC5972549

[4] D. Y. Heng , W. Xie , M. M. Regan , et al., “Prognostic Factors for Overall Survival in Patients With Metastatic Renal Cell Carcinoma Treated With Vascular Endothelial Growth Factor‐Targeted Agents: Results From a Large, Multicenter Study,” Journal of Clinical Oncology 27, no. 34 (2009): 5794–5799. 19826129 10.1200/JCO.2008.21.4809

[5] A. Crouch , A. Chaudhri , S. Khan , M. Ma , N. Vu , and W. Towers , “Acute Management of Hypercalcemia of Malignancy—A Review of Pathophysiology, Diagnosis, and Treatment,” Current Oncology Reports 27, no. 7 (2025): 871–882. 40434677 10.1007/s11912-025-01685-z

